# Use and understanding of functional cognitive disorder terminology in United Kingdom clinical practice - a survey

**DOI:** 10.1192/bjo.2021.737

**Published:** 2021-06-18

**Authors:** Alexandra Nash, Jon Stone, Alan Carson, Craig Ritchie, Laura McWhirter

**Affiliations:** Centre for Clinical Brain Sciences, University of Edinburgh

## Abstract

**Aims:**

This study aimed to explore the terms used by old-age psychiatrists and psychologists to describe subjective and mild cognitive impairment and functional cognitive disorders (FCD) in clinical practice.

**Method:**

Participants were selected from across the United Kingdom based on their clinical involvement in the assessment of cognitive complaints. 9 old-age psychiatrists and 4 psychologists were interviewed about their use of terminology in clinical practice and their awareness and understanding of FCD terminology via semi-structured interview questions and case vignettes. Interviews were conducted between December 2020 and February 2021 using online platforms Zoom and Microsoft Teams. Participants were recruited by email and Twitter. All questions were asked verbally; however, the four case vignettes were displayed via screen-share. All discussions and answers were transcribed and transcripts were coded manually using the exploratory case study methodology in order to identify themes in participants’ responses.

**Result:**

This study has highlighted the variable use of terms used to describe and diagnose patients presenting with symptoms of cognitive disorders. The terms ‘mild cognitive impairment’, ‘subjective cognitive decline’ and ‘functional cognitive disorder’ were used most commonly amongst participants, though the terms ‘subjective cognitive impairment’ and ‘pseudodementia’ were also presented. This theme of language discontinuity is underscored by participants’ varying use of terminology when describing or presenting their diagnoses for the case vignettes. The data also reveals a sub-theme of variability in application of the term FCD. Whilst all participants gave similar definitions for this term, the application of FCD as a diagnosis in practice was inconsistent. Six participants described FCD as associated with or secondary to other functional or psychiatric conditions, four participants viewed FCD as an isolated diagnosis, and one participant considered FCD to be either part of another illness or a separate diagnosis. Two participants neither used nor recognised the term FCD.

**Conclusion:**

It is evident that there is varied use of terms describing or diagnosing forms of cognitive symptoms. The findings of this study highlight the need for a clear, adoptable definition of FCD in practice as well as implementable management plans for FCD patients. This is critical in order to avoid misdiagnosis and mismanagement, which may have harmful effects on patients living with debilitating cognitive symptoms.

